# Discordant Response of Systemic Mastocytosis Associated With Myelodysplastic Syndrome After Midostaurin and Allogeneic Hematopoietic Stem-cell Transplantation

**DOI:** 10.1097/HS9.0000000000000478

**Published:** 2020-10-20

**Authors:** Marion Haas, Roch Houot, Francisco Llamas-Gutierrez, Marie-Laure Boulland, Mikael Roussel, Thierry Lamy, Thierry Fest, Cedric Pastoret

**Affiliations:** 1 Centre Hospitalier Universitaire de Rennes, Laboratoire d’Hématologie, Pôle de biologie, Rennes, France; 2 Centre Hospitalier Universitaire de Rennes, Service d’Hématologie clinique, Rennes, France; 3 Centre Hospitalier Universitaire de Rennes, Laboratoire d’Anatomopathologie, Pôle de Biologie, Rennes, France.

Systemic mastocytosis (SM) is a rare and heterogeneous disease characterized by the accumulation of neoplastic mast cells in various tissues, predominantly skin and bone marrow (BM).^1^ Thirty percent of SM is associated with a hematological neoplasm, referred to as SM-AHN, which confers a poor prognosis. The somatic *KIT* D816V mutation is a diagnostic hallmark of SM and the major therapeutic target in advanced mastocytosis. Here, we report the case of a patient with an SM associated with a myelodysplastic syndrome (MDS) who achieved discordant responses after midostaurin and allogenic hematopoietic stem cell transplantation (HSCT). We performed serial high throughput sequencing (HTS) to evaluate the responses after each line of treatment and to guide therapeutic decision making. Through these monitoring, we investigated the clonal evolution of this complex disease.

**Figure 1:**
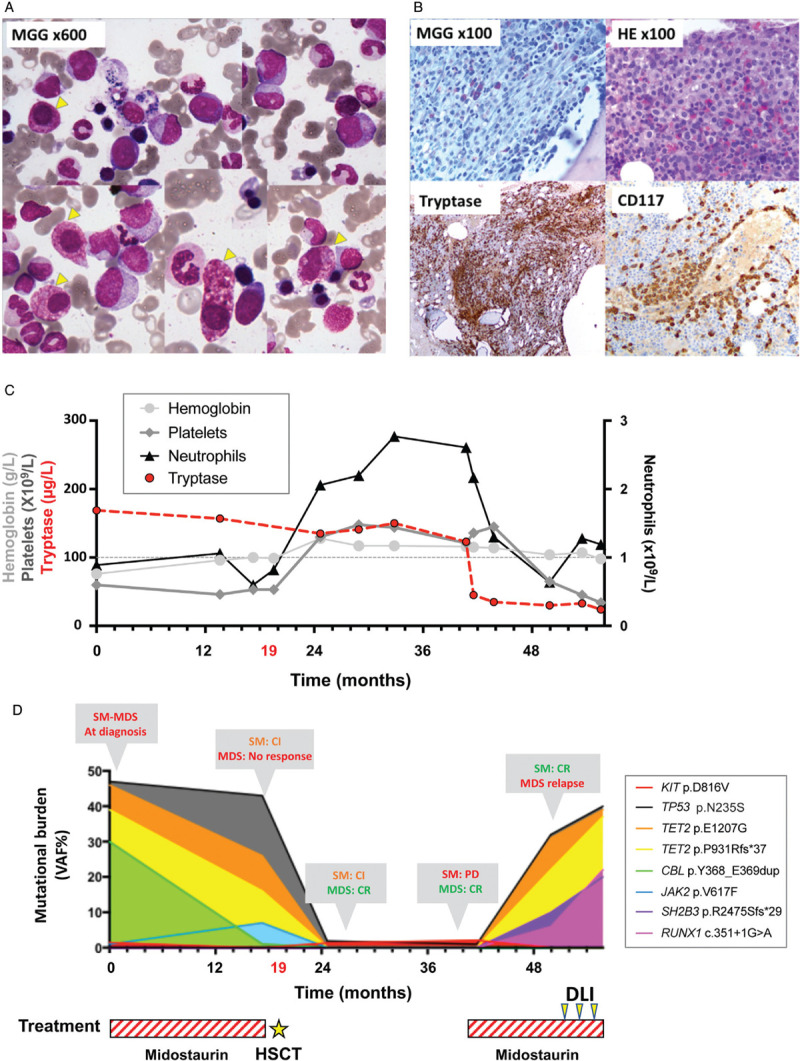
**Morphological pattern of SM–AHN and biological monitoring in regard of therapeutic options**. (A) Bone marrow (BM) aspirate smear (MGG, ×600) showing significant signs of dysgranulopoiesis with hypogranulation and marked dyserythropoiesis with nuclear atypia and laminated cytoplasm. Atypical mast cells with enlarged degranulated cytoplasm (B) BM trephine biopsy: hypogranulated and spindle shaped mast cells (HE and MGG stain, ×100); multifocal aggregates of mast cells exhibiting a strong staining for CD117(cKIT) and Tryptase. (C) Evolution of complete blood count and tryptase level. (D) Mutational burden with high throughput sequencing during the course of the disease. Treatment choice and response over successive treatment lines were reported on the mutational burden curves (CI = clinical improvement, SD = stable disease, CR = complete response).

A 59-year-old woman was referred to our Hematology department for night sweats, asthenia, weight loss, and a voluminous splenomegaly of 15 cm below the costal margin. Complete blood cell count revealed a pancytopenia (hemoglobin 7.6 g/dL, platelets 60 × 10^9^/L leukocytes 1.8 × 10^9^/L with 0.6 × 10^9^/L neutrophils) (Table 1-M0). The BM aspirate smear showed marked myelodysplastic changes in erythroid and granulocytic lineages, no ring sideroblasts, less than 5% of blasts and the presence of mast cells with abnormal cytological features: hypogranularity, oval eccentric nucleus, and spindle shape morphology (Fig. 1A). Mast cells showed a strong expression of CD25 and CD2 by flow cytometry. The BM biopsy was infiltrated by dense mast cell aggregates (more than 10 clusters > 15 mast cells) with paratrabecular and interstitial localization. They were described as degranulated, fusiform, positive for CD117, CD25, and tryptase, accounting for 10% to 15% of cellularity (Fig. 1B). Biochemical analysis revealed a normal albumin level and a grade 1 anicteric cholestasis with elevated alkaline phosphatase level at 176 UI/L (normal value below 105 UI/L). The serum tryptase level was increased to 169 μg/L (Fig. 1C) and cytogenetic analysis was normal. Finally, the CT-scan showed an osteosclerosis of L4 vertebrae and on the left pubic arch, suspect for skeletal involvement. Molecular investigations by HTS evidenced the *KIT* p.D816 V mutation at a low allelic frequency (VAF 2%), additional *TP53, TET2,* and *CBL* mutations and a subclonal *JAK2* p.V617F mutation confirmed by allele specific PCR (Fig. 1D). The diagnosis of systemic mastocytosis associated with an associated hematological neoplasm was retained (SM-AHN).

**Table 1:**
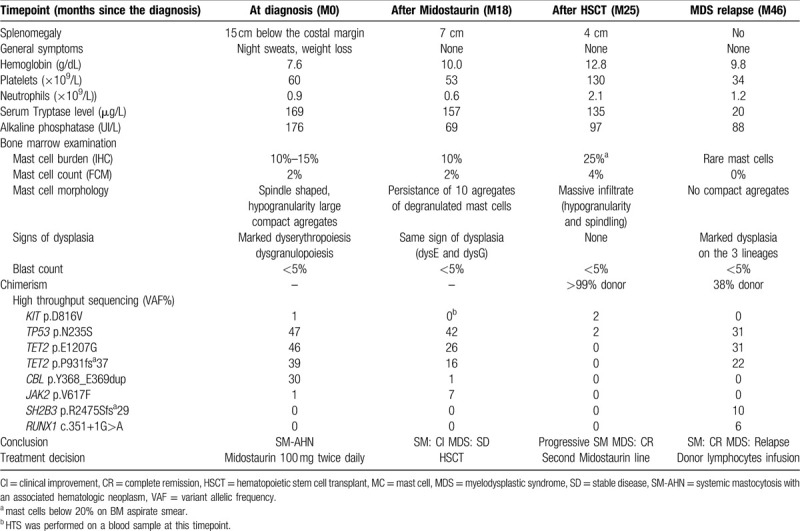
Description of Key Parameters at the Relevant Timepoints of Clinical History.

The patient received midostaurin 100 mg twice daily as front-line therapy. After one year of treatment, the patient achieved a clinical improvement according to IWG-MRT-ECNM response criteria with a reduction of the splenomegaly (>50%).^2^ The alkaline phosphatase level decreased and mast cell burden was slightly reduced. However, serum tryptase levels remained elevated. Midostaurin failed to impact the MDS compartment as cytopenias and dysplasia persisted. At this stage, the *TP53*, *TET2, CBL,* and *JAK2* mutations were still detected in the blood (Table 1M18). Consequently, the patient received an allogeneic HSCT with fludarabine, melphalan, anti-thymocyte globulin conditioning regimen. Four months after transplant, she achieved a complete response for the MDS compartment according IWG criteria with a normalized blood cell count, less than 5% of blasts, and no dysplasia features on the BM smear.^3^ Molecular investigations showed a complete donor chimerism (99%) and the disappearance of *TET2, CBL,* and *JAK2* mutations (Table 1M25). Conversely, the SM progressed after HSCT with a massive infiltration of atypical mast cells accounting for 25% of nucleated cells on BM biopsy. Moreover, *KIT* and *TP53* mutations were still detected. Thus, a second course of midostaurin was initiated and resulted in a complete remission of the SM with a decrease of tryptase levels from 130 μg/L to 20 μg/L and the disappearance of the mast cell aggregates on a new BM biopsy. Unfortunately, two years after transplant, the cytopenia reappeared and the BM smear showed new signs of dysplasia on the three lineages. Molecular investigations found an increase in the *TP53* variant allelic frequency, reappearance of the *TET2* mutations detected at diagnosis (p.E1207G and p.P931Rfs∗37) associated with new subclonal alterations of *SH2B3* and *RUNX1* genes (Fig. 1D). The chimerism decreased to 38% donor and *KIT* D816V was undetectable confirming the relapse of the MDS without evidence of SM recurrence (Table 1M46). The patient received three donor lymphocyte infusions which failed to reduce the MDS clone.

SM-AHN is the most frequent subtype of advanced SM, comprising 70% of such cases.^1^ Our report highlights the utility of HTS for the diagnosis and therapeutic management of SM-AHN. It also sheds light on the pathogenesis of this complex disease.

The diagnosis of SM-AHN is challenging. Here, the SM was confirmed by the identification of multifocal dense mast cell clusters in the BM (a major criterion in the 2016 updated WHO classification) associated with the presence of four minor SM criteria: atypical mast cells, CD2 and CD25 expression, high serum tryptase levels and presence of a *KIT* mutation.^1^ The patients presented C-findings (pancytopenia and splenomegaly) fulfilling criteria of aggressive systemic mastocytosis. However, this case also met the WHO criteria for MDS with multilineage dysplasia. Consequently, we retained the diagnosis of SM-AHN.^1^ The molecular profile at diagnosis characterized by *KIT* D816V mutation with additional MDS-related *TP53, TET2, CBL,* and *JAK2* mutations supports this diagnosis.^4^

In this report, we documented a discordant response between mast cell and MDS compartment after allogeneic HSCT and midostaurin therapy. In the literature, few articles reported similar cases.^5,6^ The most recent paper reported a complete remission of an AML immediately after HSCT and a progressive decline of neoplastic mast cells with a delay of three years.^5^ The authors discussed a late graft-versus-mast cells effect or the gradual apoptosis of mast cells due to the long lifespan of these cells. According to this report, a long follow-up is necessary before considering a treatment failure of the HSCT procedure on the clonal mast cell disease. None of the previously published reports have investigated the clonal evolution by HTS. The genomic profile of SM-AHN has been well described. The *KIT* D816V mutation is found in over 90% of the cases.^7^ Moreover, recent studies evidenced a high rate of *TET2*, *SRSF2*, *ASXL1*, *SF3B1*, *CBL*, *RUNX1*, *JAK2,* and/or *RAS* mutations.^4,8^ These additional mutations may adversely impact survival.^4^ Indeed, the Mutation-Adjusted Risk Score (MARS) integrates detection of high molecular risk mutations (*SRSF2*, *ASXL1,* and *RUNX1*) and clinical criteria (age and cytopenia) to define three-risk groups among patients with advanced SM.^9^

Progress in the understanding of the biology of advanced SM and novel molecular findings have led to the development of new therapies.^10^ Midostaurin, a multikinase/KIT inhibitor has been approved by the FDA for upfront treatment of advanced mastocytosis.^11^ Recently, the EXPLORER trial showed an overall response rate of 83% for avapritinib, a novel oral inhibitor of D816V-mutated *KIT*, which may represent a new therapeutic option for midostaurin-naive and/or refractory SM.^12^ However, the use of an allogeneic HSCT procedure remains necessary for a subgroup of patients.^13^ Their early identification is a major issue in the management of SM and could rely on clinical and molecular profile. The cumulated experience on KIT inhibitors evidences a poorer response and a reduced progression-free survival rate for SM-AHN compared to aggressive SM or mast cell leukemia.^11,14^ While the mast cell clone often responds, the response to the AHN component of the disease is more variable. Thus, SM-AHN patients may be the preferred group for consideration of HSCT, especially if the associated disorder is MDS.^13,14^ MARS intermediate- and high-risk scores may predict less robust responses with currently available therapies, including midostaurin monotherapy. Combination therapies with midostaurin and allogenic HSCT in eligible candidates should be considered for these patients.^9^

In our case, the AHN and the multiple-hit genetic profile at diagnosis were associated with an unfavorable outcome. Moreover, the serial sequencing data suggest that the MDS clone did not harbor *KIT* p.D816V, which constitutes a molecular rationale for the poor response observed on the MDS component after the first midostaurin therapy. The allogenic HSCT led to a complete response of the MDS but failed to reduce the mast cell tumor burden. The second course of midostaurin was effective in treating the SM, but the patient eventually relapsed from MDS two years after transplant. The distinct molecular profile at relapse suggests a clonal evolution of the initial MDS.

The genetic complexity of SM-AHN raises the question of the distribution and the order of acquisition of molecular alterations in mast cells and in the AHN clone. In most cases, the evidence of multilineage involvement of *KIT* p.D816V argues for an early role of this mutation in the SM-AHN pathogenesis.^15^ In such a scenario, *KIT* p.D816V is detected with a high allelic frequency and additional mutations are considered as later events in the SM pathogenesis responsible for the development of associated myeloproliferative or myelodysplastic neoplasms.^14,15^ On the other hand, Jawhar et al demonstrated that *KIT* mutations could be a distinct late event in the development of advanced SM.^8^ Thus, an initial clonal expansion at an early stage of hematopoiesis could be responsible for the emergence of a myeloid clone with MDS or MPN characteristics and the SM may arise later with the occurrence of a *KIT* mutation in a previously multi-mutated progenitor.^8^

**Figure 2:**
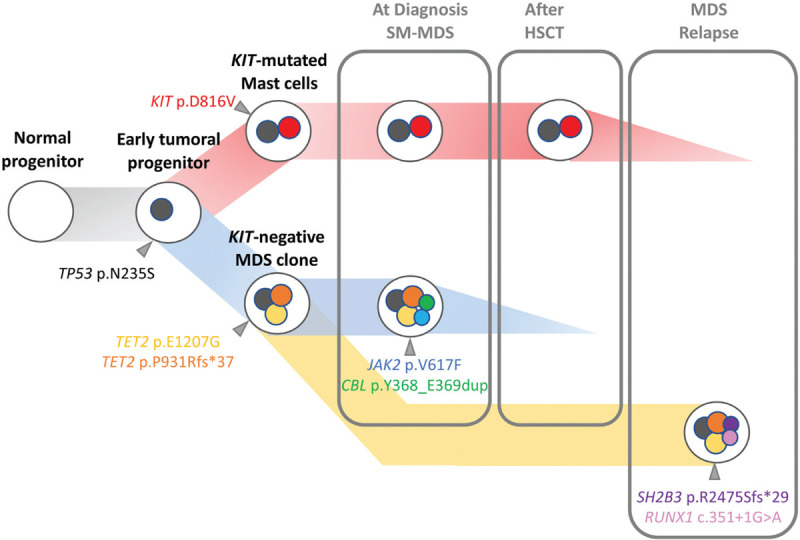
**Possible steps in the development of a SM-AHN**. Hypothetical steps in the sequential development of *KIT*-mutated systemic mastocytosis associated with a myelodysplastic syndrome in a 59-year-old woman through HTS follow-up after each therapeutic line. The *TP53* mutation (grey) was present in all bone marrow cells and probably occurred as the first event in a clonal hematopoiesis. The progeny of that cell expanded as 2 clones acquiring distinct mutations. On the one hand, the *KIT* mutation gives rise to the mast cell clone, while the myelodysplastic clone emerges with 2 *TET2* mutations (yellow and orange). Then, a dysplastic subclone arises by acquiring *JAK2* (blue) and *CBL* (green) mutations. After HSCT, only the mast cell clone persisted. After midostaurin treatment, the mast cell clone decreased while a new dysplastic subclone evolved from the *TP53* and *TET2*-mutated clone with the occurrence of a new *SH2B3* (purple) and *RUNX1* (pink) mutations.

In our report, the *TP53* mutation presented the higher allelic frequency among all detected alterations at diagnosis and this alteration persisted throughout the follow-up, arguing for an early acquisition in clonal hierarchy (Fig. 1D). Conversely, the subclonal status of the *KIT* mutation at diagnosis does not support the hypothesis of a multilineage involvement. The *KIT* D816V allelic frequency was strongly correlated with the flow cytometry-based mast cell tumor burden on BM aspirate (sample used for HTS) (Table 1). After transplantation, the patient reached a complete response of the MDS while mast cell aggregates were still detected in the BM biopsy. At this time, we evidenced that mast cells harbored both, *TP53* and *KIT* mutations. Accordingly, the *KIT* mutation was no longer detected after the second line of midostaurin (complete remission of SM and MDS relapse). This evolution confirms that the *KIT* mutation was restricted to the mast cell compartment. On the other hand, additional somatic mutations of *TET2, CBL, JAK2, SH2B3,* and *RUNX1* genes were only detected at diagnosis or MDS relapse, suggesting that they were restricted to the MDS component (Fig. 1D). Single cell sequencing of progenitor and differentiated cells derived from the SM and AHN component should be interesting to more precisely dissect the clonal hierarchy in this case. However, our serial next generation sequencing data showed that both *KIT*-mutated mast cells and the myelodysplastic clone arose from an early common *TP53*-mutated progenitor (Fig. 2). Consequently, targeting *TP53* mutant could be a relevant strategy in this case.^16^ APR-246 (Aprea Therapeutics) is the most advanced compound in clinical development, actually investigated in combination with azacytidine in patients with *TP53* mutant MDS and acute myeloid leukemia.^17^ APR-246 reactivates p53 protein function by restoring mutant p53 conformation, thereby inducing programed cell death in cancer cells.^18^

In conclusion, our case illustrates the interest of serial HTS monitoring in SM-AHN for the diagnosis, the prognostic classification and beyond for individualizing treatment strategy.
